# Facile synthesis of mesoporous nano Ni/NiO and its synergistic role as super adsorbent and photocatalyst under sunlight irradiation

**DOI:** 10.1007/s11356-022-19970-w

**Published:** 2022-04-27

**Authors:** Sahar K. Mohamed, Amira M. Elhgrasi, Omnia I. Ali

**Affiliations:** Chemistry Department, Faculty of Science, Helwan University, Ain Helwan, Cairo 11795 Egypt

**Keywords:** NiO, Ni/NiO hybrid, Adsorption, Photocatalytic degradation, Water decontamination

## Abstract

Tailoring a material that has a synergistic role as an adsorbent and a photocatalyst for environmental application is an attractive field for research. This article presents a study of facile synthesis of NiO and Ni/NiO with a synergistic role as super adsorbents in the lake of light and photocatalysts under light irradiation. Nano flower-like mesoporous NiO and Ni/NiO were synthesized by the co-precipitation method. XRD, SEM, EDAX, XPS, BET, and DR/UV–Vis spectroscopy techniques were employed for samples’ analysis. The point of zero surface charge of prepared samples was detected by the batch equilibrium method. The adsorption efficiency was investigated in the absence of light using aniline blue as a pollutant model dye. The synergistic effect as an adsorbent and a photocatalyst was investigated under UV and sunlight irradiation. Different parameters affecting the adsorption in the dark have been optimized. The results showed that in the absence of light, the prepared samples are super adsorbents with a maximum adsorption capacity ranging from 210 to 230 mg g^−1^ and a removal % ranging from 95 to 100% within 2 h. Under UV or sunlight irradiation, the adsorbent/photocatalyst attained a dye removal % of 99.8% within 30 min. The adsorption data matched the pseudo-second-order model, and the equilibrium adsorption data showed compatibility with Langmuir model. The findings of experiments revealed that the adsorption is spontaneous, exothermic, and results in less entropy. Under sunlight irradiation, the dye removal efficiency increased by 19% in the case of Ni/NiO hybrid; it showed a removal efficiency of 99.5% within 30 min under sunlight irradiation versus 80% after 120 min in the dark.

## Introduction

Transition metal oxides are interesting materials with unique optical, magnetic, and electrical properties that make them a promising candidate for unlimited applications. One of these oxides is NiO, which was applied to many applications such as memory devices, gas sensors (Tong et al. 2021), batteries (Jia et al. 2021), light-emitting diodes and lighting devices (Taeño et al. 2021b; Zhang et al. 2021), waveguides, and optical resonators (Taeño et al. 2021a). Due to its facile synthesis and stability, NiO was also examined as an adsorbent for a number of organic and inorganic pollutants such as dyes (Al-aoh 2018; Ramesh 2018), phenol (Dehmani and Abouarnadasse 2020), and heavy metals (Ziaeifar et al. 2015; Adhikari et al. 2017; Rajabi Kuyakhi and Tahmasebi Boldaji 2021). NiO is a p-type semiconductor whose band gap ranges from 3.6 to 4.3 eV and is characterized by chemical stability, low toxicity, and high ionization energy (Bonomo 2018; Taeño et al. 2021b). In the last decade, few researchers have utilized NiO as a photocatalyst for some environmental contaminants such as dyes (Jayakumar et al. 2017; Sabouri et al. 2018; Khairnar and Shrivastava 2019), antibiotics (Torki and Faghihian 2017), and free cyanide (Bashir et al. 2019).

In general, photocatalytic efficiency is enhanced by decreasing the rate of recombination of the generated electron–hole. Doping the metal oxides with elements is one of the strategies to enhance their structural and optical properties (Al Boukhari et al. 2020; Bhatt et al. 2020; Ahmed et al. 2021). The partial reduction of NiO into metallic Ni in the NiO matrix leads to the improvement of the electron–hole generation, and hence achieves a better photocatalytic activity (Paliwal and Meher 2020; Srinivasa et al. 2021). Ni/NiO hybrid was also studied as an adsorbent for Pb and Cd ions (Shivangi et al. 2020), Congo red and Cr(VI) (Zhao et al. 2016), catalytic decomposition of ozone (Gong et al. 2020), and also applied for water splitting (Wang et al. 2017). Synthesis’ conditions affect the morphology, particle size, and band gap of Ni/NiO particles (Mammadyarova et al. 2021). Ni/NiO has been fabricated by various routes, such as solution combustion using different fuels (Adhikari and Madras 2017; Srinivasa et al. 2021), thermal annealing of Ni(CH_3_CO_2_)_2_·4H_2_O and graphene mixture (Wang et al. 2021), precipitation using NaBH_4_ and ethylene glycol as a basic precipitant (Zhao et al. 2016), and precipitation of nickel oxalate in the presence of polyethylene glycol followed by calcination (Shivangi et al. 2020).

Tailoring a material that can act as an adsorbent in the lake of light and photocatalyst under light irradiation is a promising field for research (Mohamed et al. 2018; Ramesh 2018). Metal oxides that act as semiconductors could be engineered for synergistic functions as adsorbent and photocatalyst. Photocatalysis requires irradiation with light of a definite wavelength, while adsorption occurs even in the absence of light. For photocatalysis applications, different sources of light irradiation were applied as Xe lamp (Zhao et al. 2017, 2018), black light UV lamp (Abd-Rabboh et al. 2019), UV lamp (360 nm) (Mohamed and Mohamed 2018), and direct sunlight irradiation (Mohamed et al. 2018; Wang et al. 2018).

In this research, a promising approach of using the same material as an adsorbent in the absence of light and a photocatalyst under UV or sunlight irradiation was investigated. To the best of our knowledge, no previous work focused on comparing the adsorption efficiency in the dark with the photocatalytic efficiency under sunlight irradiation for NiO or Ni/NiO hybrid nanoparticles. In this article, a flower-shaped NiO was synthesized by the co-precipitation method. Ni/NiO hybrid samples were prepared via reduction reaction by glucose or urea within the calcination process. Different techniques as XRD, SEM, EDAX, XPS, DR/UV–Vis, and BET were used for samples’ characterization. Various parameters affecting the adsorption of aniline blue as a model dye were optimized. The adsorption efficiency in the absence of light irradiation was compared with the synergistic adsorption–photocatalytic efficiency under UV and direct sunlight irradiation.

## Experimental

### Materials

**Scheme 1:**
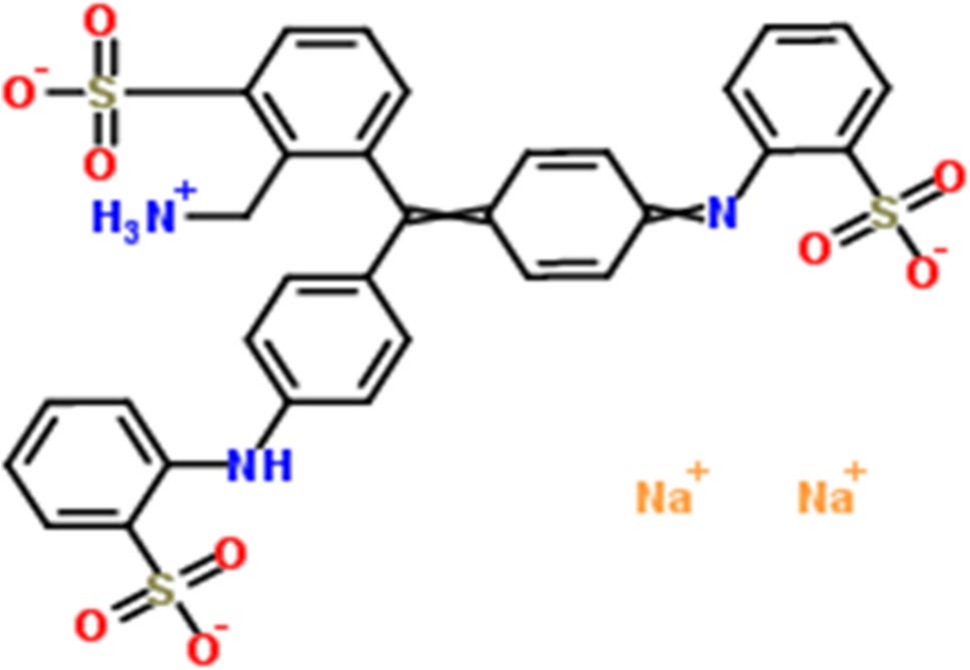
Chemical structure of aniline blue

Nickel (II) chloride hexahydrate [NiCl_2_·6H_2_O] and ammonium hydroxide (NH_4_OH) were purchased from Merck. Urea [CH_4_N_2_O] and glucose [C_6_H_12_O_6_] were obtained from ADWIC. The chemicals were used as received without further purification. Aniline blue (AB) [C_32_H_25_N_3_Na_2_O_9_S_3_] was purchased from ALPHA, UK, and its chemical structure; is shown in Scheme 1.

### Synthesis

#### Synthesis of NiO nanoparticles

Co-precipitation method was utilized to prepare NiO. Briefly, NH_4_OH was added dropwise to NiCl_2_.6H_2_O solution (10%) under constant stirring till the solution pH reached 11. The resulted mixture was aged for 24 h, then the produced light green precipitate of Ni(OH)_2_ was filtered and washed using distilled water. The resultant was dried at 70 °C then calcined at 400 °C for 4 h to get NiO nanoparticles.

#### Synthesis of Ni/NiO hybrid nanoparticles

One gram of the dried precipitate of Ni(OH)_2_ was ground in an agate mortar with either 2 g glucose or 20 g urea as reducing agents then the mixture was calcined in covered crucibles at 400 °C for 4 h. The produced fine powders were labeled as Ni/NiO_(g)_ and Ni/NiO_(u),_ respectively.

### Characterization

The structural characteristics of the samples under investigation were examined using X-ray diffractometer (X’Pert Pro, PAN analytical, NL). The X-ray source was Cu Kα radiation (*λ* = 1.5406 Å) in the 2θ range of (5° to 60°). Samples surface was explored by X-ray photoelectron spectroscopy (XPS) K-ALPHA (Themo Fisher Scientific, USA) with monochromatic X-ray Al K-alpha radiation (− 10 to 1350 eV). The samples’ morphology was explored with the field emission scanning electron microscope (FESEM) (Quanta FEG 250, the Netherlands) after coating the samples with gold. Nitrogen adsorption–desorption isotherm, pore size distribution, and surface area were explored using Quantachrome TouchWin v.1.2. The band gap values of samples were determined using UV–Vis spectrophotometer at ambient temperature (DR/UV–Vis) (Jasco–V570) in the *λ* range of 200 to 800 nm.

### Point of zero charge detection

The isoelectric point or pH_pzc_ (point of zero charge) plays a vital role in the behavior of adsorbents and photocatalysts. pH_pzc_ is the pH value at which the net charge on the dispersed phase’s surface is zero. The batch equilibrium method was employed to determine pH_pzc_ (Mohamed et al. 2019). In this experiment, a definite mass of each composition (0.05 g) was dispersed in NaNO_3_ solution (50 ml, 0.1 mol L^−1^) of definite initial pH (pH_i_) in the range of 1–10. HCl and NaOH solutions (0.1 mol L^−1^) were used to control pH_i_. After 24 h of shaking at room temperature, the final pH (pH_f_) was measured. pH_pzc_ = pH when (pH_i_ − pH_f_) equals zero.

### Adsorption studies in the lake of light (dark)

In a typical adsorption experiment, a definite mass of each sample (0.05) g was dispersed in dye solution (50 ml) of definite (*C*_*o*_), and pH was controlled by buffer solutions. The mixtures were kept under continuous stirring till equilibrium. The initial dye concentration (*C*_*o*_) and equilibrium concentration (*C*_*e*_) were measured in mg L^−1^ on a UV–Vis spectrophotometer (Jasco 730) at a wavelength (*λ*_max_) of 607 nm. The removal percentage (%*E*) and the adsorption capacity, *q*_e_ (mg g^−1^) were determined according to the following equations.

1 $$\%E=\frac{{C}_{o}- {C}_{e}}{{C}_{o}}\times 100$$

2 $${q}_{e}=\left({C}_{o}- {C}_{e}\right)\times \frac{V}{\mathrm{m}}$$

where *m* is the mass of dispersed sample (g) and *V* is the volume of AB solution (L). The effect of different parameters as pH (4–10), adsorption period (0–2 h), initial AB concentration (30–500 mg L^−1^), and temperature (30–60 °C) on the adsorption process was investigated.

For adsorption kinetics studies, each sample was dispersed in AB solution (*C*_*o*_ = 50 mg L^−1^, pH 6, 20 °C). The mixture was kept under continuous stirring in the dark, and *AB* concentration was measured at definite time intervals till equilibrium. The experimental data were examined by pseudo-first-order, pseudo-second-order, and intra-particle models.

For detection of maximum adsorption capacity and adsorption isotherms studies, the equilibrium concentration was measured for dye solutions of various *C*_*o*_ (30, 50, 130, 200, 300, or 500 mg L^−1^). The obtained data were examined using the well-known isotherms (Freundlich, Langmuir, and Dubinin–Radushkevich).

For thermodynamics studies, the adsorption experiments were repeated at different temperatures (303, 313, 323, or 333 K).

### Synergistic adsorption-photocatalytic effect under UV or sunlight irradiation

The synergistic action of the prepared samples as adsorbent and photocatalyst was examined under UV irradiation or sunlight irradiation. UV irradiation was carried out inside a homemade wooden box irradiator provided with three black light UV lamps (18 W) and a multipoint stirrer. The same experiments were conducted under direct natural sunlight irradiation during May 2020 in Cairo, Egypt where the temperature range was (35 to 40 °C). In these experiments, the sample was dispersed in *AB* solution (*C*_*o*_ = 30 mg. L^−1^) and this dispersion was irradiated for 2 h under constant stirring. Solution temperature was observed during the experiments, and the concentration of *AB* was measured at definite time intervals. The rate constant values of the photocatalytic degradation ($${k}_{pc})$$ were detected from the slope of $$l\mathrm{n}\frac{{C}_{o}}{{C}_{t}}$$ versus time (pseudo-first order):

3 $$\mathrm{ln}\frac{{C}_{o}}{{C}_{t}}={k}_{\mathit{pc}}t$$

## Results and discussion

### Characterization

#### XRD

**Fig. 1:**
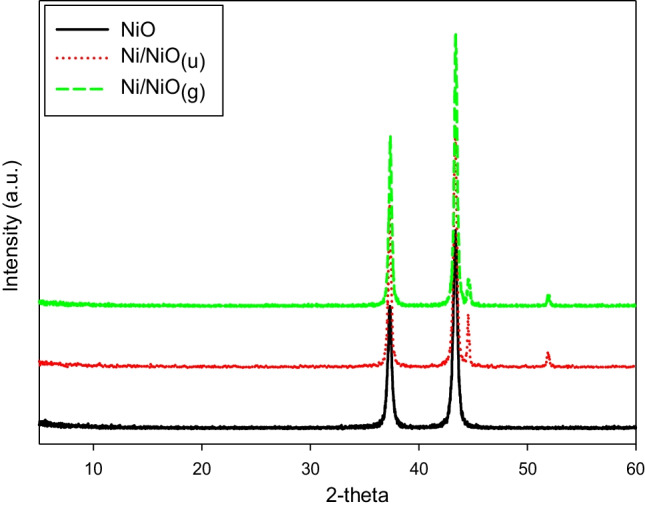
XRD analysis of NiO, and Ni/NiO hybrid samples (Ni/NiO_(g)_, Ni/NiO_(u)_)

The crystalline structure of the samples was examined by XRD, and the results are shown in Fig. 1. The XRD pattern of NiO showed sharp peaks at 2ϴ of 37.2° (111) and 43.4° (200), which indicate a face-centered cubic crystalline structure that is consistent with JCPDS card no. 47–1049 (Adhikari and Madras 2017). The samples Ni/NiO_(g)_ and Ni/NiO_(u)_ showed extra diffraction peaks at 44.5 (111) and 51.9 (200) that are characteristic of the cubic structure of crystalline Ni (JCPDS card no. 04–0850). Ni/NiO_(u)_ showed a slightly higher intensity for these peaks indicating a higher content of crystalline Ni than Ni/NiO_(g)_. The used muffle has no air input; therefore, during the calcination process, organic urea or glucose consume the available oxygen during their thermal decomposition, which leads to an oxygen deficiency in the calcination chamber. As a result, some of Ni(OH)_2_ is reduced into Ni. Utilization of 20 g of urea versus 2 g of glucose leads to a higher content of Ni in the case of Ni/NiO_(u)_ sample. Assuming a complete combustion, each 20 g of urea requires 16 g oxygen while each 2 g of glucose requires 2.1 g oxygen. These results indicate that using organic hydrocarbons during the calcination process of metal oxides or hydroxides results in the reduction of the metal to some extent.

#### SEM-EDAX analysis

**Fig. 2:**
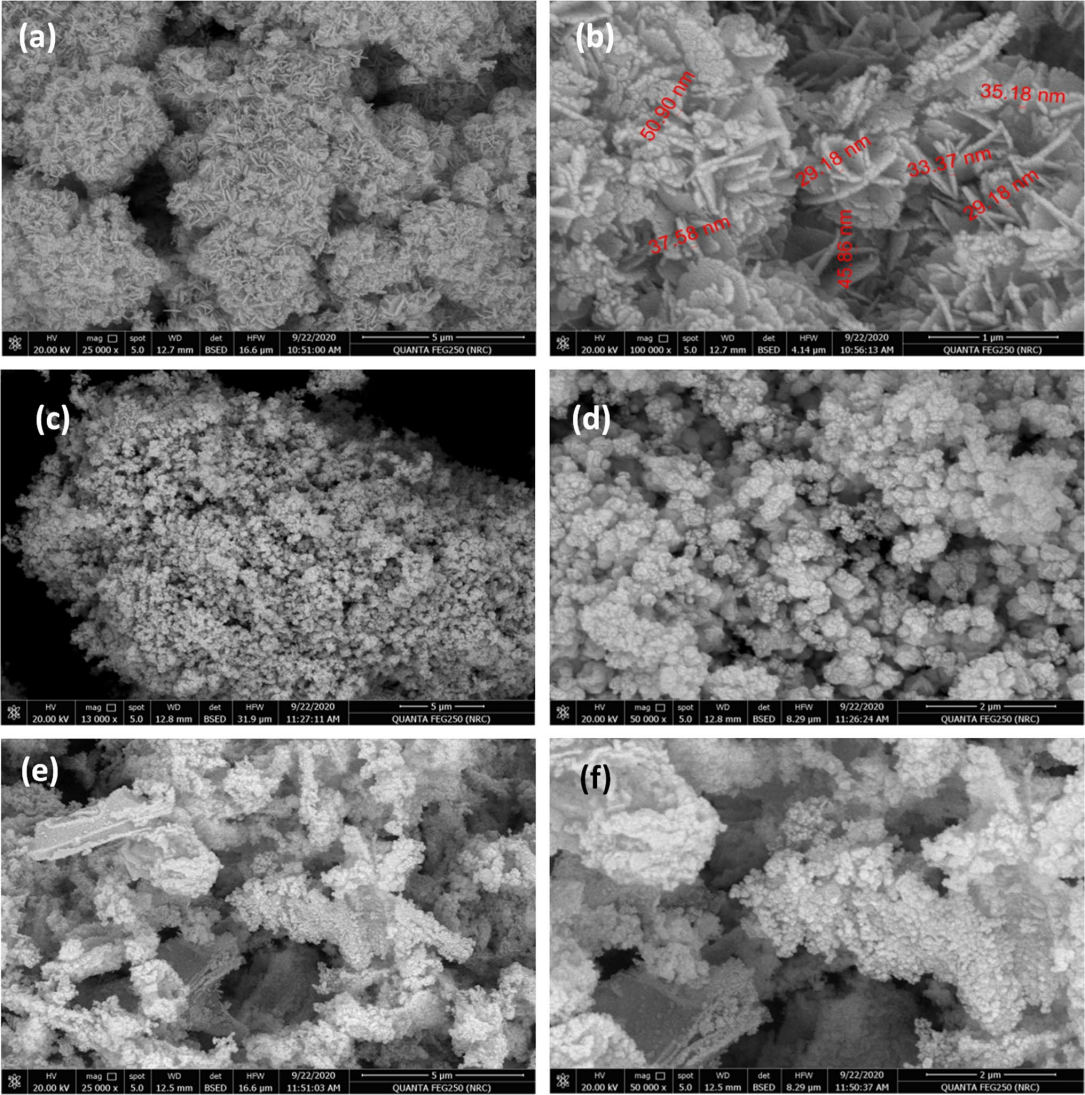
FESEM images of NiO (**a**, **b**), Ni/NiO_(g)_ (**c**, **d**), and Ni/NiO_(u)_ (**e**, **f**)

Figure 2(a−f) represents the SEM images of the samples’ surface. As shown in Fig. 2(a, b), neat NiO showed nanosheets, of a thickness ~ 29–50 nm, arranged to form a flower-shaped structure. Similar morphology was obtained for NiO prepared by hydrothermal method (Ding et al. 2016). Ni/NiO_(g)_ showed spherical shapes of polydisperse diameter forming cauliflower-like surface as displayed in Fig. 2(c, d). It seems that the released gases from the thermal decomposition of glucose suppressed the growth of the NiO nanosheets. Similar morphology was shown by Shivangi et al. when Ni/NiO was prepared by calcination of nickel oxalate in the presence of polyethylene glycol (Shivangi et al. 2020). Figure 2(e, f) exhibits agglomerates of irregular shapes with random spherical aggregates in some areas of Ni/NiO_(u)_ surface. This sample showed larger gaps appeared as channels between the agglomerates. This morphology was revealed to the generated gases from the thermal decomposition of urea that raptured the bulk during the calcination. Generally, the presence of glucose or urea during calcination significantly affected the morphology of the samples.

#### XPS

**Fig. 3:**
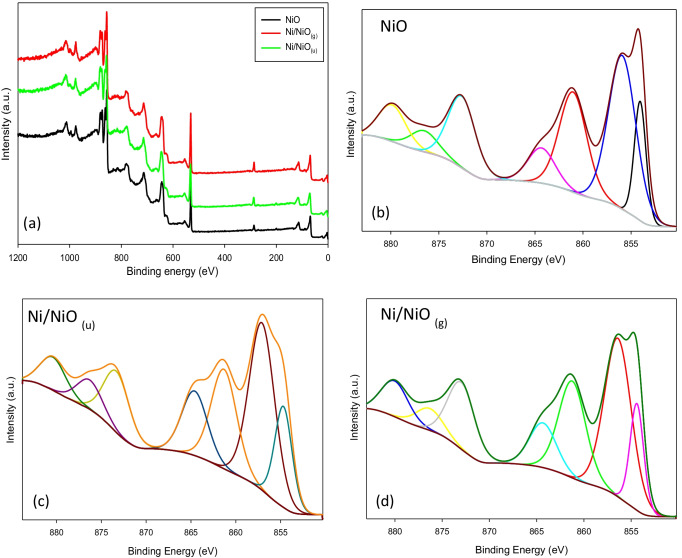
XPS survey of NiO, Ni/NiO_(g)_ and Ni/NiO_(u)_ (**a**), and Ni 2P spectra of NiO (**b**), Ni/NiO_(u)_ (**c**) and Ni/NiO_(g)_ (**d**)

XPS is used to explore the elemental composition of samples’ surfaces and the obtained spectra are presented in Fig. 3. The survey spectra (Fig. 3(a)) confirmed the presence of Ni 2p and O 1 s peaks in all samples. The spectra and fitting analysis of the three samples (Fig. 3(b−d)) showed peaks of O 1 s at 530.9 eV and the peaks of Ni 2p_3/2_ at 854.1, 855.9, 863.33, and 872.62 eV in addition to Ni 2p_3/2_ satellite at 861.04 eV. Also, extra peaks at 876.53 and 879.87 eV assigned to Ni 2p_1/2_ and Ni 2p_1/2_ satellites are shown. Although the presence of metallic Ni was confirmed by XRD results, XPS did not show clear peaks for metallic Ni (at 852.6 eV) and similar results were previously recorded (Adhikari and Madras 2017; Alam et al. 2020; Srinivasa et al. 2021). This is probably due to the nature of the XPS technique, which surveys the sample surface while metallic Ni atoms exist inside the face-centered cubic structure of NiO lattice. Moreover, no evidence for Ni^3+^ ions was shown, which eliminates the probability of Ni_2_O_3_ formation (Salunkhe et al. 2020).

#### Diffuse reflectance measurements

**Fig. 4:**
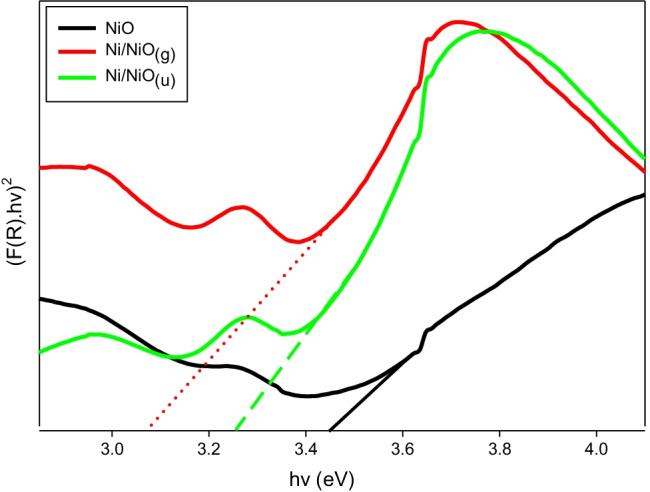
DR/UV–Vis spectra of NiO, Ni/NiO_(g)_, and Ni/NiO_(u)_

DR/UV–Vis spectrophotometer analysis is usually applied to detect the band gap energy *E*_*g*_ for semiconductors. The measurements give the reflectance as a function of wavelength *λ*. Then, the Kubelka–Munk equation is used to detect the direct optical band gap energy as follows:

4 $$F\left({R}_{\infty }\right)= \frac{{\left(1-{R}_{\infty }\right)}^{2}}{2{R}_{\infty }}$$

5 $${\left(F\left({R}_{\infty }\right). h\upsilon \right)}^{2}=A\left(h\upsilon -Eg\right)$$

where *R*_*∞*_ is the specimen’s reflectance at infinite thickness, *F(R*_*∞*_*)* is the Kubelka–Munk function, *E*_*g*_ is the energy gap, *hυ* is photon energy, and *A* is a constant. If the linear segment in the curve *of (F(R*_*∞*_*). hυ)*^*2*^ versus *hυ* is extrapolated, it touches the *x*-axis at the direct band gap energy (eV), as displayed in Fig. 4. The direct band gap energy (*E*_*g*_) values were 3.44, 3.05, and 3.25 eV for NiO, Ni/NiO_(g)_, and Ni/NiO_(u)_, respectively*.* Ni/NiO hybrid samples showed a slight red shift toward a higher wavelength (the border of visible light) (Adhikari and Madras 2017).

#### Surface area and particle size analysis

**Fig. 5:**
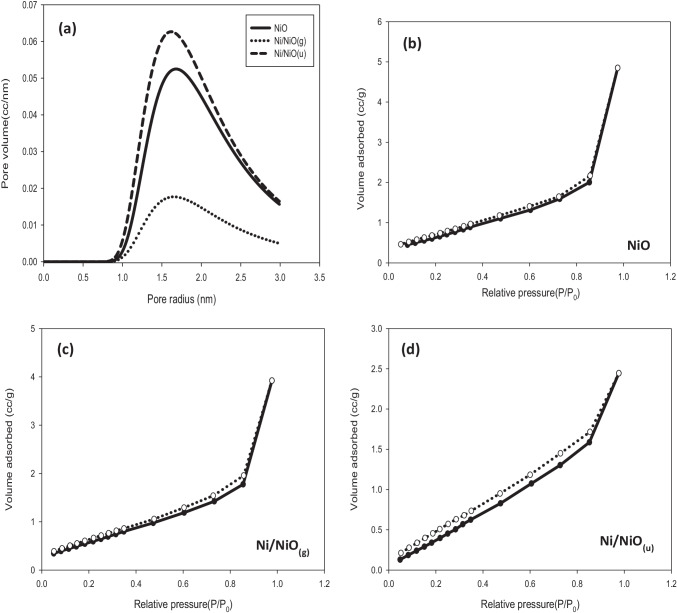
Pore size distributions of prepared samples (**a**), BET adsorption–desorption isotherms of NiO (**b**), Ni/NiO_(g)_ (**c**), and Ni/NiO_(u)_ (**d**)

It is well-known that the surface area has a vital function in the efficiency of adsorbents and photocatalysts. In the adsorption process, as the adsorbent’s surface area increases, the number of active sites also increases, and thus a greater adsorption capacity is obtained. On the other hand, photocatalysis starts with the irradiation of the catalyst surface to generate an electron–hole, and as the surface area increases, the rate of electron–hole generation increases. Table 1 shows the obtained results from BET analysis where surface area increases in the order of Ni/NiO_(g)_ < NiO < Ni/NiO_(u)_. Ni/NiO_(u)_ showed the largest surface area and the smallest average pore size, which suggests the presence of channels that are open from both sides as shown in SEM micrographs (Fig. 2(e, f)). As shown in Fig. 5(a), the samples have a broad pore radius distribution of mesoporous structure with pore radius ranges from 1 to 3 nm. It is well-known that mesoporous materials are characterized by their large surface areas, which propose the prepared samples as an excellent choice for adsorption applications. The shape of N_2_ adsorption–desorption isotherm provides information about the pore size and shape. As shown in Fig. 5(b−d), no vertical adsorption was noticed for all samples at low relative pressure indicating the lack of microporosity. All the samples exhibited isotherms of type IV with H_3_ hysteresis loops revealed to the capillary condensation where N_2_ gas molecules condense as multilayers filling mesopores. Low pressure hysteresis (relative pressure < 0.6) was observed, which suggests either volume change by swelling of some pores or cracking of the sample, or irreversible adsorption of N_2_ molecules in channels or pores of the same diameter as nitrogen molecules (Alothman 2012). In this case, cracking of the prepared sample is most probable.

**Table 1:** The Brunauer–Emmett–Teller (BET) surface area of the prepared NiO, Ni/NiO_(g)_, and Ni/NiO_(u)_ samples

Sample code	Surface area (m^2^ g^−1^)	Average particle radius (nm)	Average pore size (nm)	Total pore volume (cm^3^ g^−1^)
NiO	45.4	30.04	5.71	0.12
Ni/NiO_(g)_	16.2	84.39	5.05	0.03
Ni/NiO_(u)_	68.5	19.89	3.25	0.11

### Point of zero charge detection

**Fig. 6:**
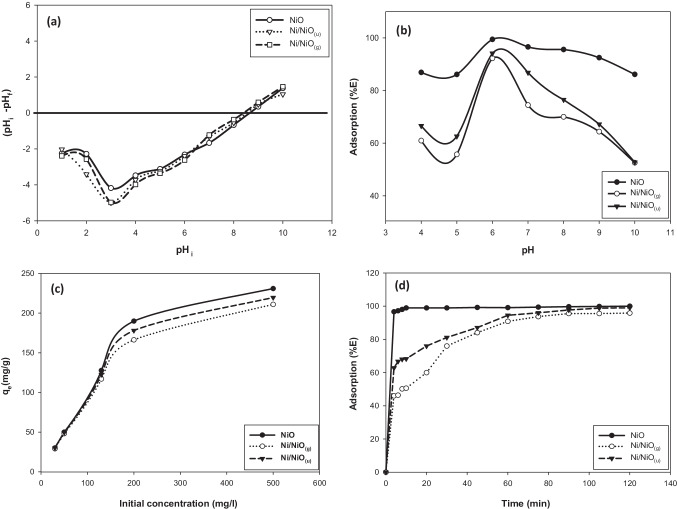
pH_pzc_ (**a**), the impact of pH on (%*E*) (**b**), the impact of initial dye concentration on adsorption capacity (**c**), and impact of contact time on (%*E*) (**d**) for NiO, Ni/NiO_(g)_, and Ni/NiO_(u)_ in the absence of any irradiation

Figure 6(a) shows the pH_pzc_ as evaluated experimentally by the batch equilibrium method. The pH_pzc_ was evaluated as 8.6, 8.3, and 8.5 for NiO, Ni/NiO_(g)_, and Ni/NiO_(u)_, respectively, that is similar to the previously published value (Mahmood et al. 2011). The presence of Ni metal did not significantly affect the surface charge of the sample. These results indicate that the net charge of the samples’ surface is positive at pH < 8.

### Adsorption studies in the lake of light (dark)

The net surface charge of the adsorbent and consequently, the adsorption process is affected by solution’s pH. Figure 6(b) represents the impact of the pH of dye solution on (%*E*). Adsorbent (0.05 g) was stirred with *AB* solution (50 ml of 50 mg L^−1^) of different pH (4–10) for 2 h at 25 °C. All samples demonstrated the best (%*E*) at pH 6. These results reveal that the adsorption of *AB* is independent of the electrostatic attraction forces. Figure 6(c) shows the impact of reaction time on (%*E*). NiO achieved 98% removal after 8 min and equilibrium was attained in 10 min. Ni/NiO_(g)_ and Ni/NiO_(u)_ showed a slower adsorption rate and attained equilibrium after 90 min. Removal (%) was 100% for NiO versus 95.8% and 99.4 for Ni/NiO_(g)_, and Ni/NiO_(u)_, respectively, which was revealed to the morphological difference between samples. In general, adsorption takes place on the surface, followed by diffusion inside adsorbent channels or pores. The maximum adsorption capacity for the samples was investigated and the results are illustrated in Fig. 6(d). Using the adsorbents for concentrated dye solutions of 500 mg L^−1^ results in *q*_*e*_ of 230, 219, and 210 mg/g for NiO, Ni/NiO_(u)_, and Ni/NiO_(g)_, respectively. NiO sample showed a faster initial rate, which revealed its morphology characteristics as it has the largest pore volume and pore size. Up to the initial dye concentration of 500 mg L^−1^, all the samples did not exhibit saturation, which indicates that the prepared samples are super adsorbents. As initial dye concentration was raised from 30 to 500 mg L^−1^, the mass transfer of adsorbate molecules from the solution to the adsorbent was enhanced thus, the adsorption capacity of NiO increased from 29.9 to 230.9 mg g^−1^.

### Adsorption kinetics

For kinetic studies, the experimental adsorption data were treated by Lagergren (pseudo-first-order), pseudo-second-order, and intra-particle diffusion models.

*Pseudo-first-order model*

6 $$\mathrm{ln}\left({q}_{e}-{q}_{t}\right)=\mathrm{ln }{q}_{e}-{k}_{1}\mathrm{t}$$

where *q*_*e*_ and *q*_*t*_ are the mass (mg) of adsorbed species per mass of adsorbent (g) at equilibrium and at time *t*, respectively. From the slope of the linear plot of ln (*q*_*e*_* − q*_*t*_) versus *t*, rate constant *k*_1_ (min^–1^) was detected.

*Pseudo-second-order model*

This equation can be expressed as follows:

7 $$\frac{t}{{q}_{t}}=\frac{1}{{k}_{2}{q}_{e}^{2}}+\frac{t}{{q}_{e}}$$

where *k*_*2*_ is the rate constant of the adsorption (g.mg^–1^ min^–1^) and is detected from the intercept of the linear plot of *t/q*_*t*_ versus *t*.

### Weber and Morris (intra-particle diffusion model)

This model is applied to explore the adsorption mechanism and clarify if the intra-particle diffusion step is the sole rate-limiting step or not. The rate constant of this step could be detected according to the following equation:

8 $${q}_{t}={k}_{i} {t}^{0.5}+C$$

The plot of *q*_*t*_ against (*t*^*0.5*^) gives a straight line of slope = *k*_*i*_ (rate constant of the intra-particle diffusion (mg g^–1^ min^–0.5^)) and intercept = *C* (parameter indicating the boundary layer effect (mg g^–1^)). As shown in Table 2, intra-particle diffusion rate constant, *k*_*i*_ increased, and boundary layer effect (*C*) decreased in the order of NiO, Ni/NiO_(g)_, and Ni/NiO_(u)_. These observations suggested that the adsorption on NiO was mainly surface adsorption while in the cases of Ni/NiO_(g)_ and Ni/NiO_(u)_, the surface adsorption was followed by diffusion of AB molecules through the pores inside the adsorbent (Pholosi et al. 2020).

**Table 2:** Adsorption kinetics data as obtained from the applied models

Adsorbent	*qe* [experimental] (mg g^−1^)	Pseudo-first order			Pseudo-second order			Intra-particle diffusion model		
		*q*_*e*_ (mg g^−1^)	*K*_*1*_ × *10*^*3*^ (min^−1^)	*R*^2^	*q*_*e*_ (mg g^−1^)	*K*_*2*_ × *10*^*3*^ (g.mg^−1^.min^−1^)	*R*^2^	*K*_*i*_ (mg/g.min^1/2^)	*C* (mg g^−1^)	*R*^2^
NiO	50.000	1.166	22.167	0.863	49.986	87.567	1.000	0.977	46.306	0.970
Ni/NiO_(g)_	47.910	43.062	55.093	0.961	51.911	2.078	0.997	2.638	26.301	0.994
Ni/NiO_(u)_	49.510	26.960	44.512	0.941	51.345	3.801	0.998	4.229	13.082	0.981

Table 2 shows that the experimental data were successfully correlated to pseudo-second-order model with the best correlation coefficients (*R*^2^), and *q*_e_ values close to experimental ones. These results suggest that the adsorption of adsorbate occurs on an energetically heterogeneous surface.

**Fig. 7:**
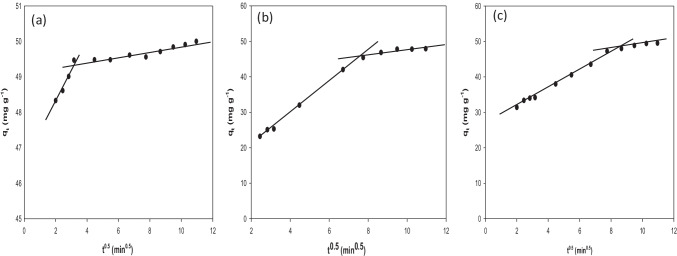
Intra-particle diffusion model’ plots of AB adsorption of on NiO (**a**), Ni/NiO_(g)_ (**b**), and Ni/NiO_(u)_ (**c**) [C_o_ of AB = 50 mg L^−1^ at 20 °C for 2 h]

Figure 7 displays the plots of *q*_*t*_ vs. (*t*^*0.5*^); the two intersecting lines over the studied time range belong to surface adsorption and diffusion through pores or channels followed by equilibrium.

The initial rate of adsorption at a time close to zero was estimated according to the following equation (Mohamed et al. 2018):

9 $${R}_{i}={k}_{2}{q}_{e}^{2}$$

Surprisingly, *R*_*i*_ values were 219.4, 4.8, and 9.3 mg/g min for NiO, Ni/NiO_(g)_, and Ni/NiO_(u)_, respectively. These results also indicate that surface adsorption is the predominant step in the case of NiO, while Ni/NiO_(g)_ and Ni/NiO_(u)_ showed a slower rate due to the slow diffusion of dye particles inside the pores and channels of the adsorbent. These findings are consistent with BET analysis results which showed that Ni/NiO_(u)_ had the largest surface area but the smallest pore size, while Ni/NiO_(g)_ had the smallest surface area and pore volume.

### Adsorption isotherms

Adsorption data at equilibrium were examined by the isotherm models of Langmuir, Freundlich, and Dubinin–Radushkevich (D-R).

*Langmuir isotherm* proposes that the adsorbent surface has a fixed number of active sites with identical activation energy to which the adsorbate molecules are attached as a monolayer neglecting the steric constraint between the adjacent adsorbed molecules (Mohamed et al. 2019). The linear form of Langmuir isotherm model is expressed as follows:

10 $$\frac{{C}_{e}}{{q}_{e}}=\frac{1}{b{Q}_{e}}+\frac{{C}_{e}}{{Q}_{e}}$$

The dimensionless constant *R*_*L*_ is calculated as follows:

11 $$ {\mathrm{R}}_{\mathrm{L}}=\frac{1}{1+{\mathrm{bC}}_{\mathrm{o}}}$$

*Freundlich isotherm* suggests the presence of active sites of dissimilar adsorption energies, on the surface of the adsorbent while the adsorbate molecules are arranged as multilayers (Freundlich 1907). The linear form of this model is written as follows:

12 $$\mathrm{log }{q}_{e}=\mathrm{log\:}{k}_{f}+\frac{1}{n}\mathrm{log\:}{C}_{e}$$

where ${Q}_{e}$ is the theoretical maximum uptake per unit mass (mg g^–1^) and *b* the Langmuir constant (L.mg^–1^). Freundlich constant (*k*_*f*_) [mg g^−1^ (mg L^−1^) ^*n*^] is related to adsorption capacity and the heterogeneity of adsorption sites energies can be indicated via the dimensionless parameter (*n*). The linear plot of log (*q*_*e*_) versus log(*C*_*e*_) is utilized to evaluate the model’s parameters (Foo and Hameed 2010).

*Dubinin–Radushkevich isotherm* model describes the adsorption on a heterogeneous system with a Gaussian energy distribution, and it is applied to investigate the adsorption mechanism (physisorption or chemisorption). This model can be presented by the following equations:

13 $$ln {q}_{e}=\mathrm{ln }{\mathrm{q}}_{\mathrm{DR}}-{\mathrm{\beta \upepsilon }}^{2}$$

14 $$\upepsilon =RT\:\mathrm{ ln}\left(1+\frac{1}{{C}_{e}}\right)$$

15 $$\mathrm{E}=\frac{1}{\sqrt{2\beta }}$$

where *q*_*DR*_ is the maximum adsorption capacity (mg·g^−1^), *β* is the Dubinin–Radushkevich constant (mol^2^. kJ^−2^), $\upepsilon$ is Polanyi’s potential, *R* is the general gas constant (8.314 J. K^−1^. mol^−1^), and *T* is the temperature (K). The numerical value *E* (mean free energy of adsorption) (kJ.mol^−1^) detects the mechanism of adsorption process (physisorption if *E* < 8 kJ.mol^−1^) or (chemisorption if 8 < *E* < 16 kJ.mol^−1^) (Hu and Zhang 2019).

Table 3 displays the calculated parameters using these models. Langmuir model offered the best fit for the experimental measurements (*R*^2^ = 0.999), suggesting a monolayer adsorption process (0 < *R*_*L*_ < 1). Dubinin–Radushkevich isotherm showed a satisfying fit, and *E* values were < 8 kJ mol^−1^ suggesting a physisorption mechanism for all samples.

**Table 3:** The obtained parameters from Langmuir, Freundlich, and Dubinin–Radushkevich (D-R) isotherm models

Adsorbent	Langmuir isotherm				Freundlich isotherm			D-R isotherm		
	*b* (L mg^−1^)	*Q*_*e*_ (mg g^−1^)	*R*_*L*_	*R*^*2*^	*K*_*f*_ [mg g^−1^ (mg L^−1^) ^n^]	*1/n*	*R*^2^	*E* (kJ mol^−1^)	*q*_*DR*_ (mg g^−1^)	*R*^2^
NiO	0.97	231.74	0.002	0.999	114.36	0.18	0.967	7.29	121.42	0.919
Ni/NiO_(g)_	0.12	216.96	0.017	0.999	37.33	0.35	0.911	0.95	142.47	0.936
Ni/NiO_(u)_	0.30	222.04	0.007	0.999	64.43	0.25	0.956	1.72	149.22	0.925

### Thermodynamics studies

Thermodynamic functions such as Gibbs energy change (Δ*G*), enthalpy change (Δ*H*), and entropy change (Δ*S*) were found from the adsorption isotherms of *AB* solutions of *C*_*o*_ = 50 mg L^–1^ for 2 at various temperatures (303, 313, 323, and 333 K). These thermodynamics parameters have been estimated from the subsequent equations:

16 $${K}_{d}=\frac{{q}_{e}}{{C}_{e}}$$

17 $$\Delta G=-RT\:\mathrm{ln} {K}_{d}$$

18 $$\mathrm{ln} {K}_{d}=\frac{\Delta S}{R}-\frac{\Delta H}{RT}$$

Table 4 represents all the thermodynamic coefficients obtained from the previous equations. The values of *R*^2^ were 0.949, 0.993, and 0.992 for NiO, Ni/NiO_(g)_, and Ni/NiO_(u)_, respectively. As shown, the negative values of Δ*H* indicate the adsorption process is exothermic, which was confirmed by the decrease in %*E* with increasing temperature. In addition, the negative values for entropy change indicate the reduction in randomness due to the arrangement of adsorbate molecules over the adsorbent surface. The spontaneity of the adsorption process of *AB* on the samples was confirmed by the negative values of Gibbs free energy. Less negative values of Δ*G* were obtained with raising the temperature from 303 to 333 K. These results reflected that the adsorption process could be suppressed with the temperature increase.

**Table 4:** Thermodynamic parameters for AB adsorption (*C*_*o*_ = 50 mg L^−1^) at pH = 6 for 2 h

T(K)	NiO				Ni/NiO_(g)_				Ni/NiO_(u)_			
	*lnK*_*d*_	*ΔG (J mol*^*-1*^*)*	*ΔH(kJ mol*^*-1*^)	*ΔS(J mol*^*-1*^ *K*^*-1*^*)*	*lnK*_*d*_	*ΔG(J mol*^*-1*^*)*	*ΔH(kJ mol*^*-1*^*)*	*ΔS(J mol*^*-1*^ *K*^*-1*^*)*	*lnK*_*d*_	*ΔG (J mol*^*-1*^*)*	*ΔH (kJ mol*^*-1*^)	*ΔS(J mol*^*-1*^ *K*^*-1*^*)*
303	2.98	− 7503.6	− 32.2	− 82.4	1.86	− 4687.8	− 30.1	− 84.0	2.02	− 5077.0	− 30.1	− 82.8
313	2.31	− 6008.0			1.39	-3627.1			1.65	− 4292.0		
323	2.13	− 5731.7			1.08	− 2910.3			1.21	− 3260.0		
333	1.76	− 4876.9			0.77	− 2137.9			0.96	− 2670.0		

### Synergistic adsorption and photocatalytic degradation processes

The synergistic adsorption–photocatalytic degradation process is a promising method that enhances the elimination of organic pollutants from their aqueous solutions. This concept targets the removal of organic pollutants using intelligent materials through an adsorption mechanism in the lake of light and a photocatalytic degradation mechanism under natural sunlight irradiation. The sunlight irradiation is available for free and supplies both ultraviolet and visible light to the earth (Lavand and Malghe 2015).

**Fig. 8:**
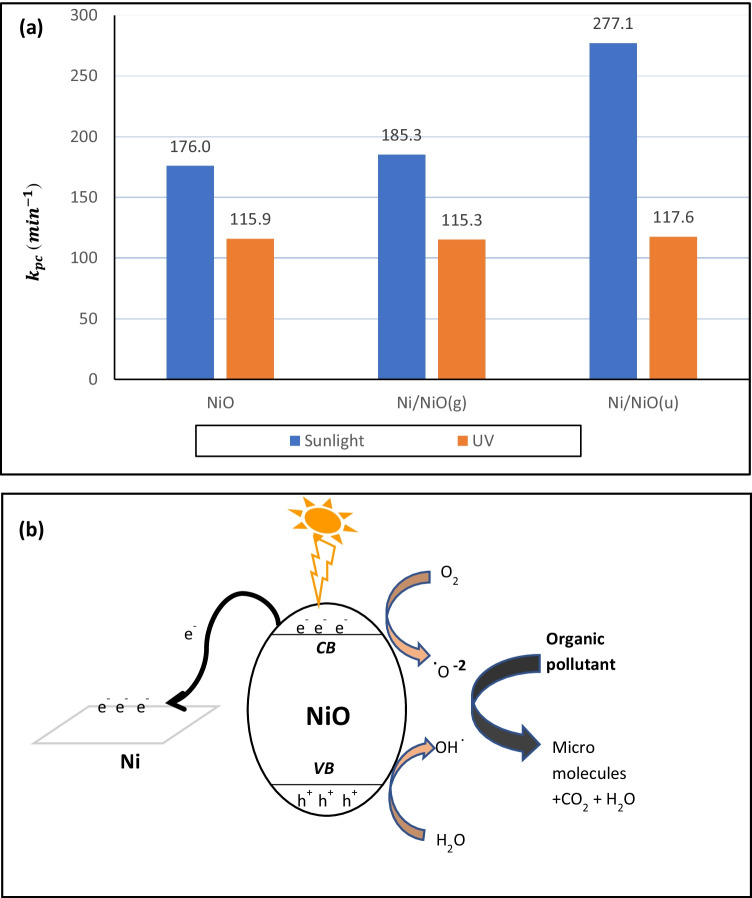
${k}_{pc}$ values for photocatalytic reaction of *AB* under UV and sunlight irradiation (**a**), and the schematic energy diagram of Ni/NiO hybrid samples (**b**)

Figure 8 presents the ${k}_{pc}$ values for *AB* dye photocatalytic degradation under UV and sunlight irradiation. All samples showed faster photocatalytic degradation under sunlight irradiation than under UV irradiation. This observation supports the fact that the prepared samples are economical candidates for the removal of organic pollutants. Using the free available sunlight instead of UV lamps save electricity. Ni/NiO hybrids had greater values for the rate constant than NiO, which indicates that the presence of metallic Ni improves the electron–hole generation or delays their recombination. Ni/NiO_(u)_ showed a faster rate than Ni/NiO_(g)_ because it has a higher content of metallic Ni as shown by XRD analysis. Srinivasa et al. stated that the enhancement of the photocatalytic efficiency of Ni/NiO revealed to the reduction in the band gap (Srinivasa et al. 2021). However, in these samples, only a slight red shift was noticed for the direct band gap energy. Herein, the enhancement in dye removal % when using Ni/NiO hybrid samples confirms the co-catalytic effect of metallic Ni, which traps the generated electrons and delays the electron–hole recombination (Fig. 8b) (Adhikari and Madras 2017; Gong et al. 2020; Zhong et al. 2020; Wojty and Baran 2021).

The photocatalytic degradation mechanism could be briefly explained as follows: when the photocatalyst’s surface is irradiated, electron–hole pairs are produced due to the migration of electrons from the valence band to the conduction band. The e^–^ converts the dissolved oxygen into (O_2_^**.−**^) while h^+^ generates (HO^**.**^) from H_2_O. These photo-generated active species and (h^+^) are accountable for the degradation of the adsorbed dye molecules according to the following equations:

$$\mathrm{phothocatalyst}+\mathrm h\mathrm\upsilon\;\left(\mathrm{irradiation}\right)\rightarrow\mathrm{phothocatalyst}\;\left(\mathrm e^-+\mathrm h^+\right)$$

$${\mathrm{e}}^{-}+{\mathrm{O}}_{2}\to {\mathrm{O}}_{2}^{\bullet -}$$

$${\mathrm{H}}_{2}\mathrm{O}+{\mathrm{h}}^{+}\to {\mathrm{HO}}^{\bullet }+{\mathrm{h}}^{+}$$

$${\mathrm{O}}_{2}^{\bullet -},+{\mathrm{HO}}^{\bullet },{\mathrm{h}}^{+}+\mathrm{dye\:molecules}\to \mathrm{micromolecules}+{\mathrm{H}}_{2}\mathrm{O}+{\mathrm{CO}}_{2}$$

**Fig. 9:**
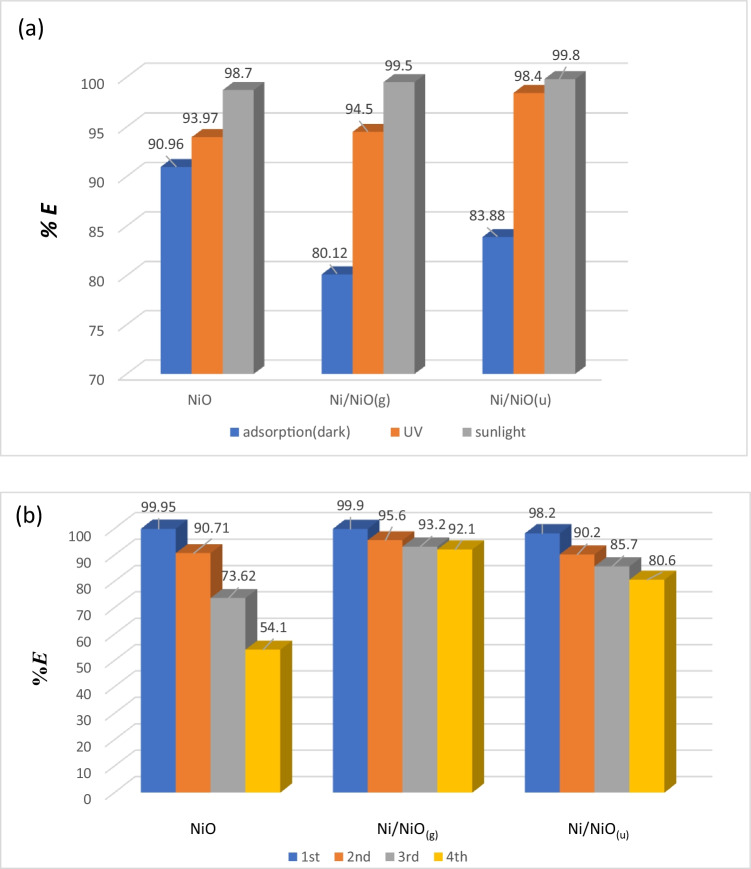
Comparison of dye removal % in the dark, under UV, and sunlight irradiation (**a**) and removal efficiency after 4 cycles (**b**)

Figure 9(a) explores comparative results of the removal % by adsorption in a dark place, without any irradiation, (measurements after 2 h) with that under UV irradiation and sunlight irradiation (after 30 min). All photocatalytic tests were done with a dye solution of *C*_o_ = 10 mg L^−1^, pH 6, and at 40 °C under continuous stirring. All samples showed the best removal % under sunlight irradiation. Ni/NiO hybrid samples showed significant enhancement in removal % (up to 19%) under irradiation. Taking into consideration that the adsorption process takes 2 h versus only 30 min for the photocatalytic degradation process, the removal % was enhanced by 8, 19, and 16% for NiO, Ni/NiO_(g)_, and Ni/NiO_(u)_, respectively, under natural sunlight irradiation. Although Ni/NiO_(g)_ has a smaller surface area (4 times less) than Ni/NiO_(u)_, its narrower band gap significantly enhanced the photocatalytic efficiency, even so it was comparable to that of Ni/NiO_(u)_. Generally speaking, the three samples successfully exhibit the synergistic role as an adsorbent in the absence of light and photocatalyst under UV or natural sunlight irradiation. In addition, the samples showed acceptable reusability after four consecutive photocatalytic cycles as explored in Fig. 9(b).

## Conclusions

The synergistic role of metal/metal oxides as an adsorbent in the lake of light irradiation and as a photocatalyst whenever light irradiation is available is a promising approach for wastewater remediation. In this study, flower-shaped nano mesoporous NiO and mesoporous Ni/NiO have been synthesized via facile co-precipitation reaction followed by partial reduction within the calcination process. The synergistic adsorption-photocatalytic role of these samples was examined using aniline blue as a model pollutant in aqueous solutions. In the absence of light irradiation, the prepared samples worked as super adsorbents where the pseudo-second-order model and Langmuir model were well correlated to the experimental data. The Dubinin model indicated the physisorption nature of the adsorption process. The adsorption occurred in a two-step mechanism, started with surface adsorption followed by the stage of diffusion. Thermodynamics investigations confirmed that the adsorption was exothermic, spontaneous, and resulted in a more ordered system. The dye removal % under sunlight was enhanced by 8, 19, and 18% for NiO, Ni/NiO_(g)_, and Ni/NiO_(u)_, respectively, within a shorter time verifying the synergistic effect. After four consecutive cycles of photocatalysis in sunlight, Ni/NiO samples were stable and efficiently reusable. The experimental data showed that the partial reduction of NiO into Ni/NiO caused a small red shift in the band gap energy and that metallic Ni has a co-catalytic effect on NiO. The synergistic role of Ni/NiO hybrid samples presents them as promising materials for wastewater remediation applications.

## Data Availability

Raw data are available upon request.

## References

[CR1] Abd-Rabboh HSM, Eissa M, Mohamed SK, Hamdy MS (2019) Synthesis of ZnO by thermal decomposition of different precursors: photocatalytic performance under UV and visible light illumination. Mater Res Express 6. 10.1088/2053-1591/ab04ff

[CR2] Adhikari S, Madras G (2017). Role of Ni in hetero-architectured NiO/Ni composites for enhanced catalytic performance. Phys Chem Chem Phys.

[CR3] Adhikari S, Madras G, Abuhatab S, El-Qanni A, Al-Qalaq H, Hmoudah MA-ZW (2017). Effective adsorptive removal of Zn^2+^, Cu^2+^, and Cr^3+^ heavy metals from aqueous solutions using silica-based embedded with NiO and MgO nanoparticles. J Environ Manage.

[CR4] Ahmed AAA, Alahsab EAA, Abdulwahab AM (2021). The influence of Zn and Mg doping on the structural and optical properties of NiO nano-structures for optoelectronic applications. Results Phys.

[CR5] Al-aoh HA (2018) Adsorption performances of nickel oxide nanoparticles (NiO NPs) towards bromophenol blue dye (BB). Desalin Water 110:229–238. 10.5004/dwt.2018.22223

[CR6] Al Boukhari J, Khalaf A, Sayed Hassan R, Awad R (2020). Structural, optical and magnetic properties of pure and rare earth-doped NiO nanoparticles. Appl Phys A Mater Sci Process.

[CR7] Alam MM, Rahman MM, Uddin MT, Asiri AM, Uddin J, Islam M (2020). Fabrication of enzyme-less folic acid sensor probe based on facile ternary doped Fe2O3/NiO/Mn2O3 nanoparticles. Curr Res Biotechnol.

[CR8] Alothman ZA (2012). A review: Fundamental aspects of silicate mesoporous materials. Materials (Basel).

[CR9] Bashir AKH, Razanamahandry LC, Nwanya AC, Kaviyarasu K, Saban W, Mohamed HEA, Ntwampe SKO, Ezema FI, Maaza M (2019). Biosynthesis of NiO nanoparticles for photodegradation of free cyanide solutions under ultraviolet light. J Phys Chem Solids.

[CR10] Bhatt AS, Ranjitha R, Santosh MS, Ravikumar CR, Prashantha SC, Maphanga RR, Silva GFB (2020). Optical and electrochemical applications of Li-doped NiO nanostructures synthesized via facile microwave technique. Materials (Basel).

[CR11] Bonomo M (2018) Synthesis and characterization of NiO nanostructures: a review. J Nanoparticle Res 20. 10.1007/s11051-018-4327-y

[CR12] Dehmani Y, Abouarnadasse S (2020). Study of the adsorbent properties of nickel oxide for phenol depollution. Arab J Chem.

[CR13] Ding C, Gao WC, Zhao Y, Zhao Y, Zhou H, Li J, Jin H (2016). Effects of Co 2+ doping on physicochemical behaviors of hierarchical NiO nanostructure. Appl Surf Sci.

[CR14] Freundlich H (1907). Ueber die Adsorption in Loesungen. H Z Phys Chem.

[CR15] Gong S, Wang A, Wang Y, Liu H, Han N, Chen Y (2020). Heterostructured Ni/NiO nanocatalysts for ozone decomposition. ACS Appl Nano Mater.

[CR16] Hu Q, Zhang Z (2019). Application of Dubinin-Radushkevich isotherm model at the solid/solution interface: a theoretical analysis. J Mol Liq.

[CR17] Jayakumar G, Albert IA, Dhayal RA (2017). Photocatalytic degradation of methylene blue by nickel oxide nanoparticles. Mater Today Proc.

[CR18] Jia X, Liu B, Liu J, Zhang S, Sun Z, He X, Li H, Wang G, Chang H (2021). Fabrication of NiO-carbon nanotube/sulfur composites for lithium-sulfur battery application. RSC Adv.

[CR19] Foo KY, Hameed BH (2010). Insights into the modeling of adsorption isotherm systems. Chem Eng J.

[CR20] Khairnar SD, Shrivastava VS (2019). Facile synthesis of nickel oxide nanoparticles for the degradation of Methylene blue and Rhodamine B dye: a comparative study. J Taibah Univ Sci.

[CR21] Lavand AB, Malghe YS (2015). Visible light photocatalytic degradation of 4-chlorophenol using C / ZnO / CdS nanocomposite. J Saudi Chem Soc.

[CR22] Mahmood T, Saddique MT, Naeem A, Westerhoff P, Mustafa S, Alum A (2011). Comparison of different methods for the point of zero charge determination of NiO. Ind Eng Chem Res.

[CR23] Mammadyarova SJ, Muradov MB, Maharramov AM, Eyvazova GM, Aghamaliyev A, Balayeva OO, Hasanova I (2021). Synthesis and characterization of Ni / NiO nanochains. Mater Chem Phys.

[CR24] Mohamed HH, Mohamed SK (2018) Rutile TiO2 nanorods/MWCNT composites for enhanced simultaneous photocatalytic oxidation of organic dyes and reduction of metal ions. Mater Res Express 5. 10.1088/2053-1591/aaa73b

[CR25] Mohamed SK, Alazhary AM, Al-zaqri NA, Alharthi A, Fahad A, Hamdy MS (2019). Cost-effective adsorbent from arabinogalactan and pectin of cactus pear peels : kinetics and thermodynamics studies. Int J Biol Macromol.

[CR26] Mohamed SK, Hegazy SH, Abdelwahab NA, Ramadan AM (2018). Coupled adsorption-photocatalytic degradation of crystal violet under sunlight using chemically synthesized grafted sodium alginate/ZnO/graphene oxide composite. Int J Biol Macromol.

[CR27] Paliwal MK, Meher SK (2020). Study of “Ni-doping” and “open-pore microstructure” as physico-electrochemical stimuli towards the electrocatalytic efficiency of Ni/NiO for the oxygen evolution reaction. New J Chem.

[CR28] Pholosi A, Naidoo EB, Ofomaja AE (2020). Intraparticle diffusion of Cr(VI) through biomass and magnetite coated biomass: a comparative kinetic and diffusion study. S Afr J Chem Eng.

[CR29] Rajabi Kuyakhi H, Tahmasebi Boldaji R (2021) Developing an adaptive neuro-fuzzy inference system based on particle swarm optimization model for forecasting Cr(VI) removal by NiO nanoparticles. Environ Prog Sustain Energy 40.10.1002/ep.13597

[CR30] Ramesh M (2018) Adsorption and photocatalytic properties of NiO nanoparticles synthesized via a thermal decomposition process. 601–610. 10.1557/jmr.2018.30

[CR31] Sabouri Z, Akbari A, Hosseini HA, Darroudi M (2018). Facile green synthesis of NiO nanoparticles and investigation of dye degradation and cytotoxicity effects. J Mol Struct.

[CR32] Salunkhe P, Muhammed Ali AV, Kekuda D (2020) Investigation on tailoring physical properties of nickel oxide thin films grown by dc magnetron sputtering. Mater Res Express 7. 10.1088/2053-1591/ab69c5

[CR33] Shivangi, Bhardwaj S, Sarkar T (2020). Core–shell type magnetic Ni/NiO nanoparticles as recyclable adsorbent for Pb (II) and Cd (II) ions: one-pot synthesis, adsorption performance, and mechanism. J Taiwan Inst Chem Eng.

[CR34] Srinivasa N, Hughes JP, Adarakatti PS, Manjunatha C, Samuel JR, Ashoka S, Craig EB (2021). Facile synthesis of Ni/NiO nanocomposites: the effect of Ni content in NiO upon the oxygen evolution reaction within alkaline media. RSC Adv.

[CR35] Taeño M, Maestre D, Cremades A (2021). Resonant cavity modes in nickel oxide microcrystals. Mater Lett.

[CR36] Taeño M, Maestre D, Cremades A (2021). An approach to emerging optical and optoelectronic applications based on NiO micro- and nanostructures. Nanophotonics.

[CR37] Tong B, Meng G, Deng Z, Gao J, Liu H, Dai T, Wang S, Shao J, Tao R, Kong F, Tong W, Luo X, Fang X (2021). Sc-doped NiO nanoflowers sensor with rich oxygen vacancy defects for enhancing VOCs sensing performances. J Alloys Compd.

[CR38] Torki F, Faghihian H (2017). Photocatalytic activity of NiS, NiO and coupled NiS-NiO for degradation of pharmaceutical pollutant cephalexin under visible light. RSC Adv.

[CR39] Wang J, Xie Y, Yao Y, Huang X, Willinger M, Shao L (2017). Ni/NiO nanoparticles on a phosphorous oxide/graphene hybrid for efficient electrocatalytic water splitting. J Mater Chem A.

[CR40] Wang L, Wang Y, Su D, Zhao Y (2018). Enhancement of visible light photocatalytic activity over bistructural SnO_2_ nanobelts. Superlattices Microstruct.

[CR41] Wang S, Xu P, Tian JJ, Liu Z, Feng L (2021). Phase structure tuning of graphene supported Ni-NiO nanoparticles for enhanced urea oxidation performance. Electrochim Acta.

[CR42] Wojty S, Baran T (2021). Copper-nickel-oxide nanomaterial for photoelectrochemical hydrogen evolution and photocatalytic degradation of volatile organic compounds. Mater Res Bull.

[CR43] Zhang Y, Dong Z, Li P (2021). Construction of electron and grain boundary barrier in quantum dots light-emitting diodes: the role of NiO interface coating. Opt Mater (Amst).

[CR44] Zhao J, Zha J, Lu H, Yang C, Yan K, Meng X (2016). Cauliflower-like Ni/NiO and NiO architectures transformed from nickel alkoxide and their excellent removal of Congo red and Cr(VI) ions from water. RSC Adv.

[CR45] Zhao Y, Zhang X, Liu J, Wang C, Li J, Jin H (2018). Graphene oxide modified nano-sized BaTiO_3_ as photocatalyst. Ceram Int.

[CR46] Zhao Y, Zhang X, Wang C, Zhao Y, Zhou H, Li J, Jin H (2017). The synthesis of hierarchical nanostructured MoS_2_/graphene composites with enhanced visible-light photo-degradation property. Appl Surf Sci.

[CR47] Zhong S, Xi Y, Wu S, Liu Q, Zhao L, Bai S (2020). Hybrid cocatalysts in semiconductor-based photocatalysis and photoelectrocatalysis. J Mater Chem A.

[CR48] Ziaeifar N, Khosravi M, Behnajady MA, Sohrabi MR, Modirshahla N (2015). Optimizing adsorption of Cr(VI) from aqueous solutions by NiO nanoparticles using Taguchi and response surface methods. Water Sci Technol.

